# Portopulmonary Hypertension and Hepatopulmonary Syndrome: Contrasting Pathophysiology and Implications for Liver Transplantation

**DOI:** 10.3390/jcm15010072

**Published:** 2025-12-22

**Authors:** Vanja Silić, Daniela Bandić Pavlović, Feđa Džubur, Ivan Romić, Igor Petrović, Goran Pavlek, Jurica Zedelj, Gzim Redžepi, Miroslav Samaržija

**Affiliations:** 1 Clinic for Anesthesiology, Reanimatology and Intensive Care, University Hospital Centre Zagreb, 10000 Zagreb, Croatia; vanja.silic@gmail.com (V.S.); dani.bandic@gmail.com (D.B.P.); 2 Clinic for Pulmonary Diseases Jordanovac, University Hospital Centre Zagreb, 10000 Zagreb, Croatia; fedja1104@gmail.com; 3 Department of Hepatobiliary and Transplantation Surgery, Clinic for Surgery, University Hospital Centre Zagreb, 10000 Zagreb, Croatia; i.romic@gmail.com (I.R.); igor.petrovic33@gmail.com (I.P.); goranpavlek@gmail.com (G.P.); jzedelj@gmail.com (J.Z.); 4 Special Hospital Primamed, 10000 Zagreb, Croatia; ravnatelj@primamed.hr

**Keywords:** portopulmonary hypertension, hepatopulmonary syndrome, liver transplantation, hemodynamics

## Abstract

Portopulmonary hypertension (PoPH) and hepatopulmonary syndrome (HPS) present two vascular complications of portal hypertension, which make opposite extremes occur against the same pathophysiological background. In PoPH, vasoconstriction predominates, along with gradual remodeling of pulmonary arteries, while HPS develops due to pathological vasodilation and creation of intrapulmonary shunts. Even though they come about by different mechanisms, both disorders significantly affect quality of life, survival, and the possibility of liver transplant. In the early phases, in clinical practice, symptoms are mainly mild and nonspecific, and overlapping with symptoms of advanced liver disease often delays forming a diagnosis. In PoPH, elevated pressures in pulmonary arteries and increased vascular resistance are observed, while HPS exhibits arterial hypoxemia with normal or lowered pulmonary pressure. Standard diagnostic workup includes echocardiography, right-heart catheterization, and analysis of the arterial gases. In patients with severe PoPH, pronounced pulmonary hypertension can represent absolute contraindication for liver transplantation due to risk of acute right heart failure during operation. Conversely, HPS usually resolves itself after a successful transplant, which confirms that the transplant is an indication of being potentially curative. Therapeutic possibilities for both states are still limited. In PoPH, specific vasodilators and supportive measures are applied, while HPS treatment is mostly supportive, directed at maintaining oxygenation until the transplant. Future research should be focused on the development of targeted therapies that address vascular remodeling, angiogenesis, and oxidative stress, as well as on the standardization of diagnostic criteria and multicentric cooperation. This approach would facilitate earlier recognition, a precise assessment of transplantability, and a better long-term outcome for patients with portal hypertension and lung vascular complications. **Key Points:** Portopulmonary hypertension (PoPH) and hepatopulmonary syndrome (HPS) represent two opposite vascular complications of portal hypertension, posing distinct challenges for liver transplantation. This review summarizes their pathophysiology, diagnostic pathways, and therapeutic strategies, emphasizing the importance of hemodynamic profiling and multidisciplinary management to optimize transplant outcomes.

## 1. Introduction

### 1.1. Consolidated Epidemiology

Portopulmonary hypertension (PoPH) and hepatopulmonary syndrome (HPS) are two vascular complications of portal hypertension that arise from a shared pathophysiological background but develop into opposite hemodynamic profiles [1,2,3,4,5]. Their reported prevalence varies depending on population, diagnostic criteria, and methodological approach. PoPH appears in approximately 2–6% of candidates for liver transplantation [1,2,3], while in the general population with portal hypertension, it is even rarer due to nonspecific early symptoms and delayed detection [2,4]. Conversely, HPS is significantly more common, with a prevalence of 10–30% among patients with advanced cirrhosis or portal hypertension [5,6,7]. Data from multicenter registries (UNOS, Eurotransplant) show that HPS accounts for 5–10% of transplanted patients, especially those receiving MELD-exception status [8,9]. Despite their divergent mechanisms—PoPH is characterized by vasoconstriction and increased pulmonary pressures, and HPS is defined by pathological vasodilation, intrapulmonary shunts, and hypoxemia—both conditions significantly influence transplant assessment and perioperative risk [1,4,6]. PoPH may delay or even contraindicate liver transplantation due to the risk of acute right ventricular failure [3,4], whereas HPS is frequently detected during routine pretransplant evaluation and typically improves or resolves after successful transplant, confirming its potential reversibility [5,8,9]. Understanding their epidemiology and clinical relevance is essential for structured diagnostic evaluation and transplant decision- making. Table 1 summarizes prevalence estimates across key patient populations.

The literature included in this narrative review was identified through a focused search of PubMed and Scopus using the terms portopulmonary hypertension, hepatopulmonary syndrome, and liver transplantation, with emphasis on studies published in the last 15 years.

### 1.2. The Pathophysiology of Portopulmonary Hypertension and Hepatopulmonary Syndrome

**Figure 1:**
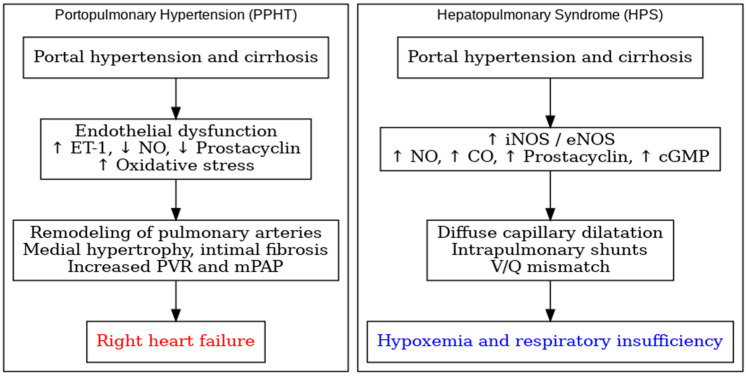
Comparative pathophysiological pathways in portopulmonary hypertension and hepatopulmonary syndrome.

Portopulmonary hypertension (PoPH) develops in the setting of portal hypertension regardless of the presence of advanced-stage cirrhosis [1,2]. Its pathogenesis involves a complex interaction of hemodynamic, molecular, and immunological factors, with endothelial dysfunction playing a central role [1,2,5]. The imbalance between vasoconstrictor and vasodilator pathways results in progressive vascular remodeling and an increase in pulmonary vascular resistance. In portal hypertension, impaired hepatic clearance of circulating vasoconstrictors further aggravates these abnormalities [4]. Endothelin receptors ETA and ETB regulate pulmonary arterial tone. Under normal physiological conditions, ETB receptors stimulate nitric oxide (NO) and prostacyclin release, leading to vasodilation. In PoPH, dominant ETA activation and reduced ETB-mediated vasodilation favor vasoconstriction, smooth muscle proliferation, and structural remodeling [5]. Reduced endothelial NO synthase (eNOS) activity further decreases NO bioavailability and eliminates its vasodilatory effect [10]. The contrasting hemodynamic and molecular pathways that distinguish PoPH from HPS are summarized in Figure 1.

Oxidative stress contributes to endothelial injury, inflammation, and vascular smooth muscle proliferation [6,7]. Inflammatory mediators, including IL-6 and TNF-α, promote endothelial activation and accelerate pulmonary vascular remodeling [8]. Translocation of bacterial endotoxins due to impaired gut–liver barrier function amplifies systemic inflammation and contributes to pulmonary vascular dysfunction [9]. In contrast, hepatopulmonary syndrome (HPS), despite occurring within the same hemodynamic context, is characterized by pathological intrapulmonary vasodilation and shunt formation, leading to ventilation–perfusion mismatch and hypoxemia. Excessive NO production due to increased inducible NO synthase (iNOS) activity is a hallmark feature, together with elevations in carbon monoxide (CO) and prostacyclin, all of which enhance cyclic guanosine monophosphate (cGMP)-mediated vasodilation [11,12]. Histologically, diffuse dilation of pulmonary capillaries—particularly in the basal lung regions—explains classic manifestations, such as orthodeoxia and platypnea [13]. Inflammatory and angiogenic pathways are also central in HPS. IL-6 and TNF-α promote angiogenesis via increased vascular endothelial growth factor (VEGF) expression [10]. These mechanisms contribute to the formation of morphologically abnormal vascular channels that facilitate intrapulmonary shunting. Experimental models show that these abnormalities can regress after liver transplantation, confirming the reversible nature of HPS [14]. Overall, PoPH and HPS represent two opposite pulmonary vascular responses to the same initiating condition—portal hypertension. PoPH involves predominant vasoconstriction and proliferative remodeling, whereas HPS is marked by excessive vasodilation, angiogenesis, and shunt formation. Despite their divergent profiles, both syndromes share a common inflammatory and endotoxin-driven microenvironment that shapes the pathological vascular response [2,9,10,15].

Although the evidence base for PoPH and HPS has expanded, most available studies remain limited by small sample sizes, heterogeneous methodologies, and reliance on observational designs. Randomized trials are scarce, and reporting quality varies, which reduces the strength and generalizability of current conclusions. These limitations should be considered when interpreting existing recommendations.

**Figure 2:**
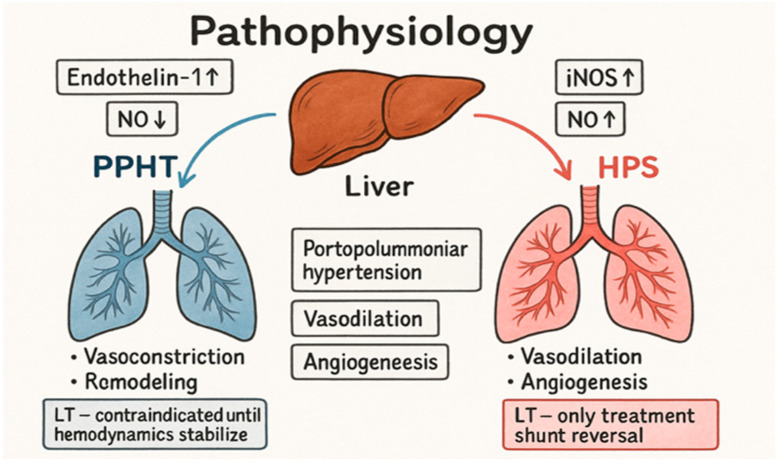
Pathophysiology of hepatopulmonary syndrome and portpulmonary hypertension.

Figure 2 illustrates how endothelial activation in portal hypertension may diverge into vasodilatation and shunting in HPS or vasoconstriction and remodeling in PoPH.

Similar effects also have other vasodilators, such as carbon monoxide (CO) and prostacyclin, whose formation also increases with the activation of cyclic guanosine monophosphate (cGMP), which enhances smooth muscle relaxation [11,12]. Histologically, in the lungs of patients with HPS, diffused dilated capillaries are observed, especially in the basal parts of lungs, which explains typical clinical manifestations, such as orthodeoxia and platypnea [10,13]. The role of inflammatory and angiogenic factors in pathogenesis in HPS is also clearly recognized: IL-6 and TNF-α activate alveolar macrophages and stimulate angiogenesis through increased expression of vascular endothelial growth factor (VEGF) [14,16]. These mechanisms jointly contribute to formation of new, morphologically and functionally abnormal vessels, which enable development of intrapulmonary capillary and intrapulmonary shunts. Experimental models confirmed that pathological changes in HPS can reversibly normalize after liver transplantation, which confirms its reversible nature [17,18,19].

**Figure 3:**
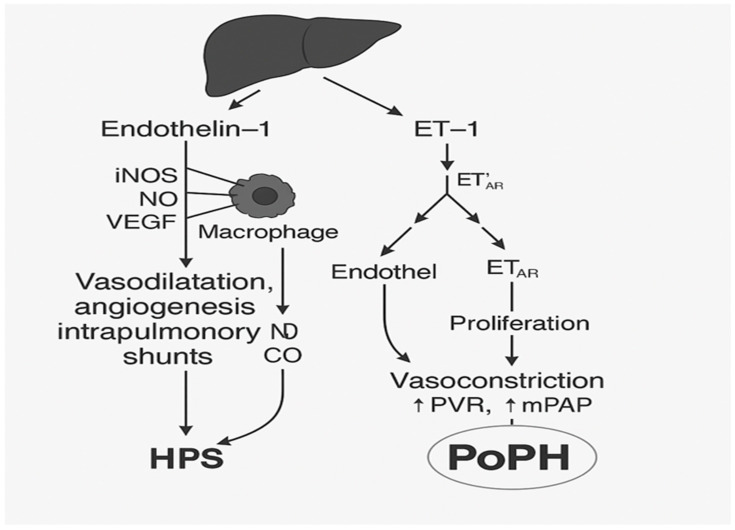
Pathophysiological mechanisms linking portal hypertension to hepatopulmonary syndrome (HPS) and portopulmonary hypertension (PoPH).

The comparison of PoPH and HPS clearly shows two opposite vascular reactions within the same clinical environment of portal hypertension: in PoPH, vasoconstriction dominates, along with proliferation and remodeling of pulmonary arteries, while in HPS, vasodilation, angiogenesis, and creation of intrapulmonary shunts combine to create conditions for severe hypoxemia and respiratory failure (Figure 3). Despite opposite mechanisms, both entities share common features: inflammatory and endotoxin-mediated microenvironments in which cytokines and endotoxins play key roles in shaping pathological response. This common inflammatory basis confirms that PoPH and HPS, although contrasting in vascular features, represent two manifestations of similar systemic dysregulation involving the liver, lungs, and immune system [2,9,10,15,20]. These mechanisms are further illustrated in Figure 2, which outlines how portal hypertension-induced endothelial activation can lead either to vasodilatation and intrapulmonary shunting in HPS or to vasoconstriction and vascular remodeling in PoPH.

## 2. Diagnosis

### Clinical Manifestations and Diagnostic Approach

PoPH and HPS can arise in the same clinical setting of portal hypertension but present with different symptom profiles and opposite hemodynamic patterns. These differences explain why timely recognition is essential when evaluating candidates for liver transplantation.

PoPH most commonly presents with nonspecific symptoms, such as reduced exercise tolerance, fatigue, mild progressive exertional dyspnea, and in advanced cases, syncope or signs of right-sided heart failure. Physical examination may reveal a loud P2 over the pulmonary artery and a systolic murmur of tricuspid regurgitation. ECG may show right ventricular strain, while chest radiography can demonstrate enlarged pulmonary arteries. Right-heart catheterization remains mandatory for confirmation of PoPH.

According to European Society of Cardiology/European Respiratory Society guidelines, the hemodynamic definition of PoPH includes the following:

- mPAP > 20 mmHg;
- PVR ≥ 2 Wood units;
- PAWP ≤ 15 mmHg, excluding postcapillary PH.

Three severity categories are distinguished:

- Mild: mPAP 20–35 mmHg, PVR < 3 WU;
- Moderate: 35–50 mmHg, PVR 3–5 WU;
- Severe: mPAP > 50 mmHg, PVR > 5 WU.

Importantly, hyperdynamic circulation in cirrhosis may elevate mPAP due to increased cardiac output, while PVR remains low; this does not constitute true pulmonary hypertension [10].

HPS presents a completely different clinical picture. The hallmark symptom is progressive dyspnea, often accompanied by the following:

- Platypnea (dyspnea worsens when upright) [4,5,9,13];
- Orthodeoxia (drop in PaO_2_ by >5% or >4 mmHg on standing) [4,5,9,13].

Additional signs may include cyanosis and spider angiomas.

Diagnosis of HPS is based on four mandatory criteria [4,5,13]:

1. Chronic liver disease with portal hypertension;
2. Arterial hypoxemia: PaO_2_ < 80 mmHg or elevated A—a gradient;
3. Proof of intrapulmonary vasodilation, usually via contrast-enhanced echocardiography (microbubble test);
4. Exclusion of alternative causes of hypoxemia.

Severity categories are as follows [4,13]:

- Mild: PaO_2_ > 80 mmHg;
- Moderate: 60–79 mmHg;
- Severe: 50–59 mmHg;
- Very severe: PaO_2_ < 50 mmHg.

The microbubble test is diagnostic when bubbles appear in the left atrium after 3–6 cardiac cycles, confirming intrapulmonary shunting [6,9,13].

Lung perfusion scintigraphy (99mTc-MAA) may demonstrate shunt if >6% of particles bypass the lungs [9,13]. Advanced techniques (dual-energy CT, perfusion MRI) can quantify abnormalities but are not yet standardized.

Differentiating PoPH vs. HPS requires strict application of standardized parameters, summarized in Table 2.

In the differential diagnosis of PoPH and HPS, it is important to exclude intracardiac shunts (e.g., PFO or ASD) and distinguish hemodynamic changes in cirrhosis from true pulmonary hypertension [1,7]. Standard protocol for the evaluation of candidates for liver transplant includes a transthoracic echocardiography in all patients, and if needed, also analysis of arterial gases and invasive methods [3,7]. A multidisciplinary team, including hepatologists, anesthesiologists, cardiologists, and pulmonologists, plays a key role in interpreting the findings and making a decision regarding transplantability [8,9]. Timely recognition of PoPH and HPS is of crucial importance, because both conditions directly determine therapeutic strategy, transplantation risk, and long-term outcomes [3,6]. Early introduction of specific pharmacological therapy directed at the stabilization of hemodynamics and the reduction of pulmonary vascular resistance is crucial in PoPH, because it enables safe liver transplant [6,7]. The recent ILTS consensus (2024) further emphasizes that structured multidisciplinary evaluation and unified diagnostic criteria between centers improve preoperative outcomes and transplant selection in both PoPH and HPS [15]. In contrast, transplantation is considered as only a curative therapy for HPS after confirmation of hypoxemia and intrapulmonary shunt [4,5]. In candidates for liver transplantation, echocardiography, arterial gas analysis, and invasive hemodynamic assessment form the diagnostic cornerstone. A multidisciplinary team is essential for interpreting findings and determining transplant eligibility. Early diagnosis is crucial because both conditions strongly influence therapeutic decisions, perioperative risk, and long-term outcomes.

## 3. Results and Treatment

### 3.1. Therapeutic Implications

The treatment of PoPH and HPS is based on completely different therapeutic strategies, which arise from their opposite pathophysiological mechanisms [1,2]. In PoPH, the main therapeutic goal is to reduce pulmonary vascular resistance and achieve hemodynamic stability, which enables safe liver transplantation [3,4]. Conversely, in HPS, liver transplantation represents the only proven causal therapy, while all other pharmacological or interventional approaches have shown limited effectiveness up to date [5]. Evidence for most pharmacological therapies in PoPH comes from small observation studies, and patient response is highly variable. The main pharmacological and supportive therapies for PoPH and HPS, including their mechanisms, clinical effects, and limitations, are summarized in Table 3.

### 3.2. Implications for Clinical Practice

Even in very severe cases, when partial oxygen pressure (PaO_2_) falls below 50 mmHg, transplantation can be considered, although perioperative risk is significantly elevated [4,5]. Therefore, MELD system includes an exception that allows patients with HPS priority access to transplantation [8]. This fundamental difference between PoPH and HPS shapes the clinical approach: in PoPH, transplants must not be performed without prior pharmacological preparation; in HPS, transplantation is indicated regardless of severity of disease, because it represents the only form of causal treatment [6,8,9]. The recent ILTS 2024 consensus further highlights the importance of early hemodynamic profiling and individualized and preoperative planning to minimize right ventricular failure risk [15]. Such polarization of therapeutic strategies emphasizes the importance of timely selection of patients, accurate diagnostic categorization, and a multidisciplinary approach, which ensures optimal preparation and safe perioperative execution of the transplant [7,8,9].

#### 3.2.1. Treatment of Portopulmonary Hypertension

Liver transplantation without prior pharmacological optimization carries high perioperative risk, primarily due to the possibility of acute right ventricular failure [6,7]. Therefore, in clinical practice, a range of drugs is applied and developed for pulmonary arterial hypertension (PAH), whose goal is to improve oxygenation and reduce vascular resistance [6,7].

The most commonly used drug groups include the following:

- Endothelin receptor antagonists (ERA);
- Phosphodiesterase type 5 inhibitors (PDE-5i);
- Prostacyclin analogs and agonists;
- Soluble guanylate cyclase (sGC) stimulators.

Typical starting doses for commonly used agents include macitentan 10 mg daily, sildenafil 20 mg, 3 times daily, and inhaled iloprost 2.5–5 mcg up to 6–9 times per day, adjusted according to tolerance. Safety considerations in cirrhosis are essential, particularly hepatotoxicity with endothelin receptor antagonists and systemic hypotension with PDE-5 inhibitors and prostacyclin analogs. Combination therapy (usually ERA + PDE-5 inhibitor, with or without prostacyclin) is increasingly used in patients with insufficient hemodynamic response to monotherapy. In selected PoPH patients being evaluated for transplantation, short-term escalation of vasodilator therapy can serve as a bridge to transplant strategy when aiming to achieve mPAP < 35 mmHg and PVR < 3 Wood units.

Among ERA drugs, bosentan and ambrisentan effectively reduce pulmonary vascular resistance, but their use is limited by hepatotoxicity, which represents a practical problem in patients with cirrhosis [6,8]. Macitentan, a third-generation antagonist, showed a more favorable safety profile and has been increasingly used in the transplant population. Among PDE-5 inhibitors, sildenafil and tadalafil improved oxygenation and hemodynamics, with good tolerability and simple dosing [6,8]. In severe cases, prostacyclin analogs such as epoprostenol, treprostinil, or iloprost are used, which significantly lower pulmonary pressure but require complex administration methods (intravenous, subcutaneous, or inhalational) [6,9]. For patients refractory to PDE-5 inhibitors, riociguat, an sGC stimulator, is used. A combined therapy (ERA + PDE-5i, with or without prostacyclin) is becoming an increasingly common approach, especially in patients with severe hemodynamic burden [6,9]. The success of treatment is visible in registries showing that patients who reach target values of mPAP < 35 mmHg and PVR < 3Wood can undergo transplant with acceptable perioperative risk and long-term survival comparable with other transplant populations [9,21,22].

#### 3.2.2. Treatment of Hepatopulmonary Syndrome

Unlike PoPH, no pharmacological therapy to date has shown a lasting effect on oxygenation nor improvement in prognosis of HPS [3,11]. Numerous agents (methylene blue, somatostatin, octreotide, pentoxifylline, antioxidants, and anti-inflammatory drugs) have been tested but their benefits were transient and clinically insufficient [3,11]. Interventional methods such as transjugular intrahepatic portosystemic shunt (TIPS) showed contradictory results and have not become part of standard therapy [9]. Supportive measures, including oxygen therapy, control of ascites, and optimization of volume status, remain the basis of treatment until transplantation [3,5,23,24]. Liver transplantation remains only the causal and curative approach [5]. After transplant, the majority of patients gradually show a regression of intrapulmonary shunts and normalization of oxygenation within six to twelve months, with long-term survival comparable with other transplant groups [5,9,25].

### 3.3. Liver Transplantation and Outcomes

PoPH and HPS have completely different mechanisms in a transplantation context, and decision about transplantability depends primarily on the hemodynamic status of the patient [1,6,7,10,26]. According to current guidelines, liver transplantation can be considered in patients with a mean pulmonary artery pressure (mPAP) < 35 mmHg and pulmonary vascular resistance (PVR) < 160 dyn·s·cm^−5^ (≈2 Wood units), with a preserved right ventricular function [7]. Patients with mPAP between 35 and 50 mmHg can be candidates if their PVR is lower than 160 dyn·s·cm^−5^ and right ventricular function stable. In case of mPAP > 50 mmHg or PVR > 5 Wood units, the transplant is considered contraindicated due to the extremely high risk of perioperative right ventricular failure [6,7,27]. Despite strict selection, there is still a perioperative risk in PoPH that remains significant, with mortality that can reach up to 50% in untreated patients [6]. Multicentric studies, however, have shown that in adequately treated patients, the survival rate after transplantation reaches 85% in the first year, while in those with severe forms (mPAP > 50 mmHg), the outcome is significantly worse [6,9]. In more patients, pulmonary pressures gradually improve within 6–12 months after transplantation, allowing dose reduction or discontinuation of specific therapy [5,9]

#### 3.3.1. Hepatopulmonary Syndrome

Unlike PoPH, HPS is considered a clear indication for liver transplant, independent of the degree of hypoxemia [4,5]. Although severe forms (PaO_2_ < 50 mmHg) carry increased perioperative risk, transplant is still associated with favorable long-term outcomes [4,5]. UNOS and Eurotransplant systems recognize HPS as the basis for MELD exception points, which provides patients with documented hypoxemia (PaO_2_ < 60 mmHg) and proven intrapulmonary vasodilation faster access to transplantation [8,9]. Outcomes after transplants are generally very good: the majority of patients achieve normalization of oxygenation within 6 to 12 months, and five-year survival is about 70–75%, which is comparable with other transplantation indications [4,5,9,27]. The largest challenge is the group with severe hypoxemia, who previously had perioperative mortality rates of 20–30%, but newer series show significantly better results due to more precise selection and a multidisciplinary approach [5,9]. Registries confirm that a significant portion of patients after a transplant show complete reversibility of hemodynamic changes, which additionally confirms the role of transplant as a potentially curative approach [9,28].

#### 3.3.2. Registry Data

Analysis of data from the UNOS database showed that patients with HPS constitute 5–10% of all transplanted patients, and MELD exception points significantly shortened the time to transplantation [4,8,9]. Long-term survival in this group is not worse compared to other indications. In PoPH, however, registries confirm that outcomes are extremely poor in untreated patients, while pharmacological preparation significantly improves transplantability and survival [6,9]. Data from Eurotransplant indicate that PoPH constitutes about 6% of transplant indications, with long-term outcomes comparable with control groups if timely hemodynamic optimization is performed [6,9,29]. Nevertheless, approaches to treatment and selection criteria differ among centers, which further emphasizes the importance of standardized evaluation and multidisciplinary decision-making [6,8,9].

### 3.4. Clinical Challenges and Implications

Liver transplantation in patients with PoPH and HPS carry specific perioperative challenges, which arise from different pathophysiological mechanisms of these conditions [1,5,30]. In PoPH, the main risk represents acute decompensation of right ventricle and cardiovascular collapse, especially during anhepatic phase and graft reperfusion, when a sudden increase in pulmonary vascular resistance occurs [6].

Therefore, the following are crucial:

- Precise hemodynamic monitoring;
- Timely application of inhalational vasodilators, such as nitric oxide or prostacyclin;
- Careful titration of inotropic drugs and vasopressors.

The anesthesiologist plays a key role, as intraoperative management directly influences transplant safety [1,6]. In HPS, on the other hand, the main problem is severe hypoxemia, which additionally worsens during graft reperfusion due to worsening intrapulmonary shunt and circulatory instability [5,9]. Therefore, it is necessary to ensure optimal oxygenation and use individualized ventilation strategies adapted for each patient [5,9]. In the early postoperative period, intensive monitoring is necessary because respiratory complications are more frequent than in the general transplant population. Optimal care in both entities requires close cooperation of multiple specialties, including hepatologists, cardiologists, pulmonologists, anesthesiologists, and transplantation surgeons. Such a multidisciplinary approach reduces risk and improves outcomes, especially in borderline patients, for whom decisions about transplants are the most demanding [6,9].

### 3.5. Case Examples

#### 3.5.1. Case Example 1

A male in the sixth decade of life with alcoholic cirrhosis was referred for an evaluation for a liver transplant. His cardiological history included chronic heart failure with an ejection fraction of 40% and persistent atrial fibrillation, with repeated episodes of decompensation. Right heart catheterization revealed moderate portopulmonary hypertension assessment with mean pulmonary arterial pressure (mPAP) of 32 mmHg, pulmonary vascular resistance (PVR) of 1 Wood unit, and a cardiac index of 3.5 L/min/m, suggesting a predominantly hyperdynamic circulatory pattern. Despite a low MELD score of 11 and a Child–Pugh class A, he was assigned MELD exceptional points due to the presence of portopulmonary hypertension. After careful preload optimization, diuretic titration, and rate control with beta-blockers, the patient successfully underwent an orthotopic liver transplant with favorable perioperative stability (MAP 75 mmHg, mPAP <30 mmHg) and orderly recovery.

#### 3.5.2. Case Example 2

The second patient, also in the sixth decade of life, was admitted due to decompensated cirrhosis and significant right ventricular strain. Transthoracic echocardiography revealed right ventricular dilatation (TAPSE 14 mm, RV fractional area change of 28%) and moderate tricuspid regurgitation. Invasive hemodynamic assessment confirmed mean pulmonary artery pressure (mPAP) of 29 mmHg, a preserved cardiac index of 4.1 L/min/m^2^, and low PVR (1 Wood unit). The biochemical MELD score was 14, classified as Child–Pugh class B. Due to pulmonary vascular complication, he was assigned exceptional points for a priority transplant. After intensive preoperative optimization, including diuretic therapy and stabilization of the right ventricle, the patient successfully was transplanted with a intraoperative mean arterial pressure maintained above 70 mmHg and mPAP below 30 mmHg. Postoperatively, he was extubated within 12 h, required no vasopressors after day 1, and showed complete hemodynamic recovery with normalization of right ventricle size and function by postoperative day 10. At the 3-month follow-up, he remained clinically stable (NYHA class I), with normal gas exchange and resolution of portal hypertension.

## 4. Discussion

Though much is understood in pathophysiology and therapeutic possibilities, portopulmonary hypertension (PoPH) and hepatopulmonary syndrome (HPS) are still some of the toughest challenges in transplant medicine. Data from UNOS, Eurotransplanta, and available multicenter studies demonstrate that, with timely identification and appropriate preparation, long-term outcomes can be approximated to have results similar to other transplantation populations. However, perioperative risks remain high due to differing hemodynamic profiles of these conditions. With PoPH, there is a threat of acute right ventricular decompensation, while in HPS, severe hypoxemia and intrapulmonary shunt additionally worsen circulatory instability. These risks emphasize the importance of accurate preoperative assessment and intensive intraoperative monitoring. Precisely titrating vasodilators and inotropic drugs in PoPH and maintaining adequate oxygenation in HPS is important. The coordinated work of specialists, including anesthesiologists, hepatologists, cardiologists, pulmonologists, and transplant surgeons, would be optimal. Most of the evidence, especially for HPS, often comes from smaller retrospective studies. This makes it difficult to come to a clear conclusion. The new ILTS consensus from 2024 emphasizes the need for early detection of hemodynamically risky patients, clearer definition of diagnostic criteria, and greater alignment among centers [8,9,11].

With PoPH, the importance of targeted pharmacological preparation increasingly stands out, especially with drugs that act on endothelial dysfunction, oxidative stress, and angioproliferative pathways [5,6].

The introduction of newer drugs, such as macitentan and riociguat, and the development of angiogenesis modulators open up the possibility of stabilization of hemodynamics before transplantation [6,7]. However, heterogeneity response remains a limiting factor [6,7], which further emphasizes the need for individualized therapy. In HPS, pharmacological possibilities remain modest [3,11], and previous attempts with different substances, including methylene blue, somatostatin, and antioxidants, have not indicated lasting clinical benefit. Liver transplantation continue to be the only feasible option, resulting in frequent gradual withdrawal of the intrapulmonary shunt and normalization of oxygenation within 6 to 12 months [5,9].

In clinical practice, several areas remain insufficiently defined [4,5,6]. The optimal timing for initiating pharmacological therapy in PoPH is still debated, particularly in patients with borderline hemodynamics (mPAP 35–45 mmHg) [4,5,6], where centers vary between early combined therapy and watchful optimization. Management of severe HPS with PaO_2_ < 44 mmHg also differs across transplant programs, mainly regarding prioritization and perioperative risk assessment. Post-transplant medication strategies likewise vary, especially regarding the decision for whether pulmonary vasodilators should be continued or tapered after hemodynamic improvement [4,5,6]. Further development will require more precise diagnostic methods, especially in quantification perfusion disorders and shunts, including 3D echocardiography, perfusion MRI, and SPECT with 99mTc-MAA [10,12,13]. It is also important to note the progress of identification biomarkers, which could help in earlier diagnosis or monitoring therapeutic responses.

International standardization and the cooperation of registries such as Eurotransplanta and UNOS can reduce variability among centers and enable better selection of candidates [8,9]. Overall, PoPH and HPS require well-timed recognition. This includes individualized therapeutic approaches and interdisciplinary cooperation in order to reduce perioperative risk and improve long-term outcomes. Future research directed at new therapeutic targets, optimized hemodynamic protocols, and standardized diagnostic criteria are the key to improving complex patient care.

## 5. Conclusions

Portopulmonary hypertension (PoPH) and hepatopulmonary syndrome (HPS) are two significant vascular complications of portal hypertension, which markedly affect morbidity, mortality, and the outcome of a liver transplant [1,5]. Although they arise in similar clinical environments, two opposite pathophysiological processes are indicated [1,2,3]. PoPH is marked by vasoconstriction and proliferative remodeling of pulmonary arteries, which leads to a rise in pulmonary vascular resistance and encumbrance of the right ventricle [1,2]. Contrarily, HPS is a consequence of pathological dilation in pulmonary capillaries and the formation of intrapulmonary shunts, as well as a dominant vasodilatory and angiogenic mechanism, which results in severe hypoxemia [3,11]. Here, the diagnostic approach must be clearly differentiated [4,6].

For PoPH, diagnosis relies on invasive hemodynamic evaluation of the right area of the heart, while for HPS, diagnosis is based on established arterial hypoxemia and the presence of an intrapulmonary shunt, most often through contrast echocardiography or perfusion scintigraphy [4,6,11]. Proper classification crucial is for selection therapy and assessment of transplant eligibility [6]. In PoPH, pharmacological optimization with the aim of achieving targeted hemodynamic values is a prerequisite for safe transplant. Most clinical experiences are collected with endothelin receptor antagonists, PDE-5 inhibitors, prostacyclin analogs, and sGC stimulators [6,7,8,9]. These improve hemodynamics and the functional status of the patients, although transplant is still considered as the only lasting treatment option.

In HPS, standard pharmacological therapy has no proven effect on survival. Transplant remains the only definitive treatment, with an improvement of hypoxemia within 6 to 12 months following the procedure [5,9,11]. Despite progress, the outcomes and risks still depend on timely recognition of the disease, standardized diagnostics, and individualized perioperative approaches. The development of new targeted therapies focused on endothelial dysfunction, oxidative stress, and angioproliferative mechanisms could improveme hemodynamic stability before transplantation and reduce the occurrence of perioperative risk [7,12]. Further progress will come with better standardization of criteria, expanded international cooperation, and registry-led approaches [8,13]. This will reduce the variability among centers and enable optimal care for patients with PoPH and HPS [8,9].

## Figures and Tables

**Table 1:** **Prevalence of portopulmonary hypertension and hepatopulmonary syndrome.** Summary of the reported prevalence of PoPH and HPS across different populations, showing their impact on liver transplant candidacy.

Population	PoPH (%)	HPS (%)	Source/Comment
General population with portal hypertension	2–5	10–30	PoPH is rare, HPS is a more frequent complication
Liver transplant candidates	5–10	15–20 (up to 30)	Key group, since these entities affect transplantability
UNOS database (USA)	~5 among recipients	5–10 among recipients	HPS candidates receive MELD exceptions
Eurotransplant data	<5	~6 of all indications	Data from multicenter registries
Multicenter studies	—	10–20 of listed candidates	High-risk group with progressive hypoxemia

**Table 2:** Diagnostic criteria for PoPH and HPS. Key diagnostic parameters, mandatory findings, and threshold values to define PoPH and HPS in clinical practice. Adapted from ESC/ERS 2022 and ILTS Consensus 2024 [10,15].

Condition	Mandatory Criteria	Key Investigations	Thresholds/Findings	(Optional) Shunt Quantification
PoPH	Portal hypertension (with or without cirrhosis)	Right-heart catheterization; Echocardiography.	mPAP > 20 mmHg; PVR ≥ 2 Wood units; PAWP ≤ 15 mmHg; Elevated RVSP or RV strain.	—
HPS	Chronic liver disease + portal hypertension	Arterial blood gas analysis; Contrast-enhanced transthoracic echocardiography (microbubble test).	PaO_2_ < 80 mmHg or A–a gradient ≥ 15 mmHg (≥20 mmHg if >64 y); Delayed microbubbles in left atrium (3–6 cardiac cycles).	99mTc-MAA scan: extrapulmonary uptake > 6%

**Table 3:** Therapies for PoPH and HPS. Overview of pharmacological and supportive treatment for PoPH and HPS, outlining differences in therapeutic strategy and limitations.

Condition	Therapy Class	Agents	Effects/Limitations
PoPH	1. Endothelin receptor antagonists	Macitentan (first-line, PORTICO trial, Lancet Respir Med 2019), Bosentan, Ambrisentan	↓ mPAP, improved RV function and tolerance; possible hepatotoxicity and drug interactions
	2. Prostacyclin analogs	Epoprostenol, Iloprost, Treprostinil	↓ PVR, improved RV function; complex delivery, risk of hypotension
	3. PDE-5 inhibitors	Sildenafil, Tadalafil	↓ mPAP, improved exercise tolerance; pulmonary hypotension, limited data
HPS	Oxygen therapy	Supplemental O_2_	↑ PaO_2_, symptomatic relief; no effect on disease course
	Experimental therapies	NO inhibitors, somatostatin, steroids	Tested in small studies; no proven benefit

## Data Availability

No new data were created or analyzed in this study.

## Abbreviations

The following abbreviations are used in this manuscript:

PoPH	Portopulmonary hypertension
HPS	Hepatopulmonary syndrome
ETA	Endothelin A
ETB	Endothelin B
NO	Nitric oxide
